# Lake Ohrid’s tephrochronological dataset reveals 1.36 Ma of Mediterranean explosive volcanic activity

**DOI:** 10.1038/s41597-021-01013-7

**Published:** 2021-09-02

**Authors:** Niklas Leicher, Biagio Giaccio, Giovanni Zanchetta, Roberto Sulpizio, Paul G. Albert, Emma L. Tomlinson, Markus Lagos, Alexander Francke, Bernd Wagner

**Affiliations:** 1 Institute of Geology and Mineralogy, University of Cologne, Cologne, Germany; 2 Istituto di Geologia Ambientale e Geoingegneria, CNR, Rome, Italy; 3 Istituto Nazionale di Geofisica e Vulcanologia, Rome, Italy; 4 Dipartimento di Scienze della Terra, University of Pisa, Pisa, Italy; 5 Dipartimento di Scienze della Terra e Geoambientali, University of Bari, Bari, Italy; 6 Istituto per la Dinamica dei Processi Ambientali (IDPA), CNR, Milan, Italy; 7 Department of Geography, Swansea University, Swansea, SA2 8PP UK; 8 Research Laboratory for Archaeology and the History of Art (RLAHA), University of Oxford, Oxford, OX1 3QY UK; 9 Department of Geology, Trinity College Dublin, Dublin, Ireland; 10 Institute of Geosciences and Meteorology, University of Bonn, Bonn, Germany; 11 Department of Earth and Environmental Sciences, University of Adelaide, Adelaide, Australia

**Keywords:** Volcanology, Geochemistry, Sedimentology, Natural hazards, Stratigraphy

## Abstract

Tephrochronology relies on the availability of the stratigraphical, geochemical and geochronological datasets of volcanic deposits, three preconditions which are both often only fragmentary accessible. This study presents the tephrochronological dataset from the Lake Ohrid (Balkans) sediment succession continuously reaching back to 1.36 Ma. 57 tephra layers were investigated for their morphological appearance, geochemical fingerprint, and (chrono-)stratigraphic position. Glass fragments of tephra layers were analyzed for their major element composition using Energy-Dispersive-Spectroscopy and Wavelength-Dispersive Spectroscopy and for their trace element composition by Laser Ablation-Inductively Coupled Plasma-Mass Spectrometry. Radiometric dated equivalents of 16 tephra layers and orbital tuning of geochemical proxy data provided the basis for the age-depth model of the Lake Ohrid sediment succession. The age-depth model, in turn, provides ages for unknown or undated tephra layers. This dataset forms the basis for a regional stratigraphic framework and provides insights into the central Mediterranean explosive volcanic activity during the last 1.36 Ma.

## Abstract (summary)

**Table:** 

Measurement(s)	major and minor element composition • trace element composition
Technology Type(s)	EPMA-WDS • SEM-EDS • LA-ICP-MS
Sample Characteristic - Environment	lake sediment

Machine-accessible metadata file describing the reported data: 10.6084/m9.figshare.14635578

## Background & Summary

Terrestrial sediment archives documenting the environmental evolution over long periods are scarce, but are of great importance for our understanding of environmental changes including climate change^1–4^ or the frequency of natural hazards^5^. One of these rare archives is Lake Ohrid, documenting the environmental history of the Central Mediterranean region for more than 1.36 Ma^2,6^. Lake Ohrid’s age and richness in endemic species^7^ have been of great scientific interest since the early 20^th^ century (cf. Stankovic^8^). In the framework of the International Continental Scientific Drilling Program (ICDP), the sediments of Lake Ohrid were extensively explored within the project Scientific Collaboration on Past Speciation Conditions in Lake Ohrid (SCOPSCO)^2,9,10^. The project’s major targets were exploring the age and origin of Lake Ohrid, the regional seismotectonic history, the Quaternary volcanic activity and climate change of the central Mediterranean region, as well as triggers of biological evolutionary patterns and endemic biodiversity^2,6^.

**Fig. 1:**
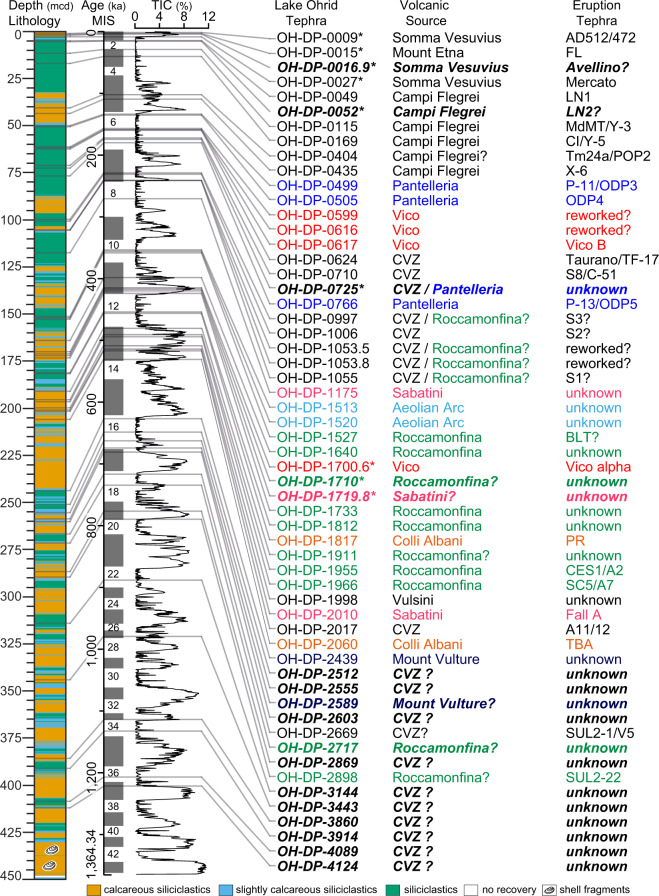
Tephrostratigraphy of the DEEP site. Lithology of the upper 450 meter composite depth (mcd) of the DEEP site from Lake Ohrid and the position of tephra layers (black bars) along with the Marine Isotope Stages (MIS, numbers of glacials given only) boundaries of Lisiecki and Raymo^59^ and the Total Inorganic Carbon (TIC) content vs. age of the DEEP site sediments^2^. High TIC contents correlate with interglacials, thus illustrating the climatostratigraphic position of tephra layers^21^. The volcanic source and equivalent eruption of tephra layers are given as published in^2,14,20,22,23,30,60^. For new tephra layers (in bold italics) the supposed volcanic origin is given according to the CaO/FeO vs. Cl diagram by Giaccio, *et al*.^61^. Tephra layers with an * represent cryptotephra. The different colors used for labelling the tephra layers correspond to their different volcanic origins. Campanian Volcanic Zone (CVZ) according to Rolandi, *et al*.^62^. Masseria del Monte Tuff (MdMT) according to Albert, *et al*.^63^. CI = Campanian Ignimbrite.

A 584 m long sediment succession (5045-1, DEEP) retrieved from the lake centre^2,9,10^ was studied to address the project aims including tephrostratigraphy and tephrochronology. Both methods rest upon the widespread dispersal of volcanic fragments (tephra) by explosive volcanic eruptions, which are quasi-simultaneously deposited in different sedimentary archives downwind of the volcano and can be identified and correlated by their unique geochemical fingerprint^11^. Tephrostratigraphy and –chronology have been proven as valuable tools for stratigraphic correlation and dating of sediment records^12^, which led to the establishment of a consistent tephrostratigraphic framework in the Mediterranean region of the last ca. 200 ka^13–19^. To extend this framework, 57 tephra layers of the DEEP site (Fig. 1) were characterized by their morphological and glass geochemical properties^2,14,20–23^. Results build on previous studies of shorter sediment successions from Lake Ohrid^24–28^, but also incorporate new stratigraphic results back to 160 ka^14,20,23^. The identification of older eruptions, of which proximal equivalents are well-dated, provided additional tephrochronological information mainly for the time period 410-530 ka^22,23^ and for the interval 720-775 ka^2^. Tephra ages, complemented by orbital tuning tie points, were used to establish a chronology of the upper 447 meter composite depth (mcd) of the sediments^2^. The age-depth model dates the lake formation at 1.36 Ma^2^ and provides the basis for the interpretation of environmental and biological processes derived from the sediment record^2,9,20,21,29^.

The geochemical fingerprint of all tephra layers analyzed indicate an origin from the Italian volcanic provinces^2,23,30^, which are located between 420-900 km upwind of Lake Ohrid and have been persistently active during the Quaternary^31^. However, knowledge especially of their Middle and Late Pleistocene volcanic history is incomplete, because proximal volcanic records are often fragmentary due to erosion and/or burial by the succeeding volcanic activity. Therefore, long and continuous distal archives, such as Lake Ohrid, can be essential to contribute to the integrity of the history of explosive volcanism by revealing previously unknown eruptions, which is demonstrated by the large number of newly discovered tephra layers in the DEEP site sequence^30^.

The dataset presented here combines individually published datasets, extends this data by additional measurements and includes new data of tephra layers not described before (Table 1). The geochemical characterization by major, minor and trace element glass compositions of tephra layers is complemented by chronological constraints from the established age-depth model^2^. In addition, morphological information such as type, thickness and color of the layers are provided.

**Table 1:** DEEP site tephra overview.

Tephra	TAS	Age ± 2σ [ka]	SEM-EDS (Pisa)	EPMA-WDS (IGAG)	EPMA-WDS (UoC)	LA-ICP-MS (iCRAG TCD)	LA-ICP-MS (UoB)	References datasets published		Extension of published datasets by the given type of data
OH-DP-0009*	pt-tp-p	1.47 ± 0.04	yes	no	yes	no	no	^20^		EPMA-WDS
OH-DP-0015*	tra-btra	3.29 ± 0.08	yes	no	yes	no	no	^20^		EPMA-WDS
OH-DP-0016.9*	btra-tp-tra-p-tr, r	3.97 ± 0.12	yes	no	yes	no	no	*this study*		
OH-DP-0027*	p	8.56 ± 0.2	no	yes	no	no	no	^23^	^20^	no
OH-DP-0049*	tr	14.57 ± 0.85	yes	no	yes	no	no	^20^		EPMA-WDS
OH-DP-0052*	tr	15.54 ± 0.99	yes	no	no	no	no	*this study*		
OH-DP-0115	tr-p	29.03 ± 0.77	no	yes	no	no	no	^23^		no
OH-DP-0169	tr-p	40.27 ± 0.34	no	yes	no	no	no	^23^		no
OH-DP-0404	p-tr-tra-tp	102.11 ± 3.09	no	yes	no	no	no	^23^		no
OH-DP-0435	tr-p	109.45 ± 1.82	no	yes	no	no	no	^23^		no
OH-DP-0499	tr-r	133.66 ± 2.89	no	yes	no	no	no	^23^		no
OH-DP-0505	tr-r	135.36 ± 4.06	yes	yes	yes	yes	no	^30^		EMPA-WDS + SEM-EDS
OH-DP-0599	p	156.89 ± 3.79	yes	no	yes	no	no	^30^		EMPA-WDS + SEM-EDS
OH-DP-0616	p-tr	158.76 ± 3.83	no	yes	no	no	no	^30^		SEM-EDS
OH-DP-0617	p	158.89 ± 3.81	no	yes	no	no	no	^30^		SEM-EDS
OH-DP-0624	pt-p	159.71 ± 4.03	no	yes	no	yes	no	^23^	^14^	no
OH-DP-0710	tp-lat	172.26 ± 5.55	yes	no	yes	no	no	^30^		EMPA-WDS + SEM-EDS
OH-DP-0725*	tr-p + r	174.44 ± 5.22	yes	no	no	no	no	*this study*		
OH-DP-0766	tr-r	180.02 ± 4.14	yes	no	yes	yes	no	^30^		SEM-EDS
OH-DP-0997	p-tr	228.87 ± 5.66	yes	no	yes	yes	no	^30^		SEM-EDS
OH-DP-1006	p-tr	230.93 ± 6.27	yes	no	yes	yes	no	^30^		EMPA-WDS + SEM-EDS
OH-DP-1053.5	p-tr	240.93 ± 6.45	yes	no	yes	no	no	^30^		SEM-EDS
OH-DP-1053.8	p-tr	240.99 ± 6.41	yes	no	yes	no	no	^30^		EMPA-WDS + SEM-EDS
OH-DP-1055	p-tr	241.23 ± 6.18	yes	no	yes	yes	no	^30^		EMPA-WDS + SEM-EDS
OH-DP-1175	p-tr	270.64 ± 4.88	yes	no	yes	yes	no	^30^		SEM-EDS
OH-DP-1513	r	353.43 ± 7.49	yes	no	yes	yes	no	^30^		SEM-EDS
OH-DP-1520	d-r	355.76 ± 7.64	yes	no	yes	yes	no	^30^		SEM-EDS
OH-DP-1527	p-tr	358.25 ± 4.64	yes	yes	yes	yes	no	^30^		EMPA-WDS + SEM-EDS
OH-DP-1640	p-tr	398.37 ± 5.95	yes	no	yes	yes	no	^30^		EMPA-WDS + SEM-EDS
OH-DP-1700.6*	r	414.75 ± 3.17	yes	yes	yes	no	no	^22^		EMPA-WDS + SEM-EDS
OH-DP-1710*	tr-p	417.13 ± 4.20	no	no	yes	no	no	*this study*		
OH-DP-1719.8*	p	419.76 ± 5.41	no	no	yes	no	no	*this study*		
OH-DP-1733	tr	423.93 ± 6.43	yes	yes	yes	no	no	^30^	^61^	SEM-EDS
OH-DP-1812	tra-p-tr	453.95 ± 2.94	yes	no	yes	yes	no	^30^		SEM-EDS
OH-DP-1817	f	456.19 ± 3.3	yes	yes	no	no	no	^23^		SEM-EDS
OH-DP-1911	p	480.49 ± 6.84	yes	no	yes	yes	no	^30^		SEM-EDS
OH-DP-1955	p-tra-pt	490.67 ± 3.92	yes	yes	no	no	no	^23^		SEM-EDS
OH-DP-1966	tra-p- tr	494.05 ± 4.43	yes	no	yes	yes	no	^30^		SEM-EDS
OH-DP-1998	p	508.67 ± 4.14	yes	no	yes	yes	no	^30^		SEM-EDS
OH-DP-2010	tp-p	514.17 ± 4.37	yes	yes	no	no	no	^23^		SEM-EDS
OH-DP-2017	tr	516.87 ± 5.47	yes	yes	no	no	no	^23^		SEM-EDS
OH-DP-2060	f	530.86 ± 3.35	yes	yes	no	no	no	^23^		SEM-EDS
OH-DP-2439	pt-tp-p	626.87 ± 3.98	yes	yes	yes	yes	no	^30^		SEM-EDS
OH-DP-2512	tr	648.56 ± 6.76	yes	no	yes	no	yes	*this study*		
OH-DP-2555	ph	662.55 ± 7.16	yes	no	yes	no	yes	*this study*		
OH-DP-2589	tp/ph	674.23 ± 5.93	yes	no	yes	no	yes	*this study*		
OH-DP-2603	tr	680.85 ± 4.46	yes	yes	yes	no	yes	*this study*		
OH-DP-2669	tr	716.74 ± 4.78	yes	yes	yes	no	yes	^2^		EMPA-WDS + SEM-EDS + LA-ICP-MS
OH-DP-2717	tp/ph	734.42 ± 5.86	yes	yes	yes	no	yes	*this study*		
OH-DP-2869	ph/tr	776.51 ± 5.14	yes	no	yes	no	yes	*this study*		
OH-DP-2898	tr/ph	789.67 ± 3.45	yes	yes	yes	no	yes	^2^		EMPA-WDS + SEM-EDS + LA-ICP-MS
OH-DP-3144	tr	888.18 ± 5.28	yes	no	yes	no	yes	*this study*		
OH-DP-3443	ph	979.33 ± 6.19	yes	no	yes	no	yes	*this study*		
OH-DP-3860	r	1113.68 ± 7.36	yes	no	yes	no	yes	*this study*		
OH-DP-3914	tr	1132.58 ± 4.27	yes	no	yes	no	yes	*this study*		
OH-DP-4089	tr	1206.9 ± 4.55	yes	no	yes	no	yes	*this study*		
OH-DP-4124	r	1221.58 ± 6.49	yes	no	yes	no	yes	*this study*		

Overview of tephra layers found in the DEEP site sediment succession providing information about the general glass geochemical composition according to the Total alkali vs. silica classification by Le Bas, *et al*.^39^, the corresponding age according to the age-depth model^2^ and the respective geochemical analyses performed at the different laboratories. Tephra layers described for the first time are marked by “this study”. If data of tephra layers was published elsewhere, the respective reference is given and it is indicated if published datasets were extended by additional SEM-EDS and/or EPMA-WDS data in this study. For tephra layers OH-DP-2669 and OH-DP-2898 trace element concentrations published in Wagner, *et al*.^2^ were recalculated using the same data reduction process as for the other samples measured at the University of Bonn^45^. d = dacite; tra = trachyandesite; tp = tephriphonolite; pt = phonotephrite; lat = latite; f = foidite; btra = basaltic trachyandesite; p = phonolite; tr = trachyte; r = rhyolite.

The combined and extended data represents a unique dataset in the Mediterranean and can be used for a variety of different applications of paleoenvironmental and volcanological studies. The detailed geochemical fingerprints and chronological information of tephra layers can be used for chronological constraints of other records in the Mediterranean region, particularly for older sediment records, which lack applicable and independent dating methods. Further, the given data also enables correlation and synchronization of records independent of their individual age-depth models and is thus ideal for exploring lead and lag relationships of climatic variability in different environments, such as demonstrated for the beginning of MIS 5^32^. The geochemical and chronological data of newly discovered tephra layers (eruptions) will help to improve the tephrostratigraphic framework and to complete our understanding of the explosive volcanic history, especially for the so far underexplored period beyond 0.6 Ma. It further provides new insights into the petrogenetic evolution of the source volcanoes and the assessment of tephra dispersal and composition-frequency-magnitude relations.

## Methods

### The DEEP site sediment succession

Seismic investigations of the ca. 30 km long and 15 km wide Lake Ohrid (Albania/North Macedonia) indicate an undisturbed sediment succession in the center of the lake^33^, where also the oldest sediments were preserved. At the DEEP site (41°02’057” N, 020°42’054” E, 243 m water depth) six parallel bore holes with an average spacing of approximately 40 m were drilled in 2013 using the Deep Lake Drilling System operated by DOSECC^9,21^ (Drilling, Observation, and Sampling of the Earth’s Continental Crust). Based on the visual core descriptions and X-ray fluorescence (XRF) downcore data of all cores, a spliced composite profile (5045-1) was established with a total length of 584 mcd^2,21^. The entire lithological succession shows two different evolutional stages of the lake. The lower interval (584-447 mcd, 79.3% recovery) is characterized by coarse-grained sediments and highly compacted peat deposits, indicating dynamic environments with fluvial to slack water conditions during the lake establishment^6^. The upper 447 mcd (100% recovery) consists of fine-grained hemi-pelagic muds that document continuous sedimentation with no evidence of unconformities or erosion, representing persistent lacustrine conditions^2^. Tephra layers were only observed in the upper 447 mcd and were used for developing an age-depth model^2^, following the approach established for the upper 247 mcd^21^. Tephra ages were imported from dated equivalents and used as chronological first order tie points after their quality was carefully reassessed^2^. Individual tephra ages also verified tuning of climate-sensitive proxy data (minima in total organic carbon; 480-year data point resolution) against orbital parameters (inflection points of increasing local summer insolation and winter-season length)^2,21^. In total, the age-depth model relies on Bayesian modelling of 16 tephra ages and 66 tuning tie points using Bacon 2.2^34^, and was cross-evaluated by the paleomagnetic ages of the Matuyama-Brunhes reversal and the base of the Jaramillo subchron^2^.

### Tephrostratigraphic methods

**Fig. 2:**
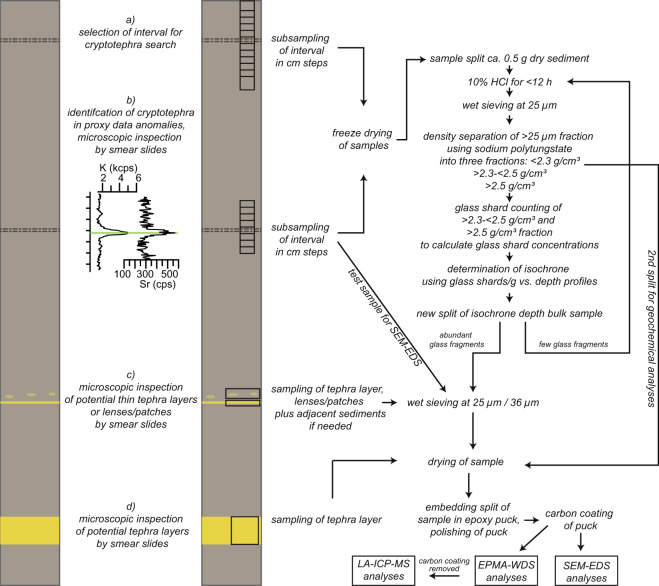
Sampling and analyses scheme of tephra layers. The sampling of tephra layers was adapted to their appearance and recognition (**a**–**d**). Cryptotephra, which was not indicated by sediment proxy data, was detected by sampling of intervals determined based on stratigraphic or chronological assessment (**a**). Cryptotephra, which was detected by anomalies in sediment proxy data, was treated as illustrated in (**b**). Thin tephra layers and lenses or patches of tephra were sampled as illustrated in (**c**). Massive tephra layers were sampled as shown in (**d**). The treatment of cryptotephra is adapted from the protocol of Blockley, *et al*.^35^.

The methods applied for sampling, processing and single-grain geochemical analysis of new tephra layers followed those as already published^2,14,20,22,23,30^. A general overview of the working scheme is given in Fig. 2 and is summarized below. Sample specific details are given along with the geochemical analyses in the main dataset.

#### Sampling and preparation of tephra layers

Sediment cores of the DEEP site were screened for macroscopic tephra layers during visual core description and subsequently using high-resolution line scan images at the University of Cologne. Smear slides of identified potential tephra layers, e.g. characterized by prompt visual grain size or color changes, were investigated by polarization microscopy (Leitz DM EP and Zeiss AxioLab A1). If volcanic fragments were identified, a bulk sediment sample covering the full thickness of the respective horizon was taken. Coarse grained samples were oven-dried (T <50 °C), whereas samples containing larger portions of fine grained material or surrounding lacustrine sediments were freeze-dried to prevent baking. If tephra horizons appeared very thin (<10 mm), as lenses, or were affected by bioturbation, an aliquot of the respective bulk sample was sieved at 25 or 36 µm to remove fine grained lacustrine sediments, enrich the volcanic fragments and to increase efficiency of subsequent single-grain geochemical analyses.

Selected intervals of the DEEP site sediment succession were also inspected for the presence of cryptotephra horizons using anomalies in downcore sediment proxy data^14,20,23^. Characteristic peaks in X-ray Fluorescence (XRF) downcore data (e.g., K, K/Ti, Sr, or Zr) obtained on an ITRAX Scanner^20,21^ (Cox, Sweden) or prompt abnormalities in the grain size distribution were tested for the presence of glass shards or micropumice by smear slides analysis. If volcanic fragments were found, for specific cryptotephra horizons, glass shard concentration profiles of respective intervals were performed to confirm their respective isochrone positions. For this purpose, profiles covering the characteristic XRF peak area and adjacent sediments above and below (<10 cm each) were sampled at 1-2 cm resolution and freeze-dried for sample processing following the protocol of Blockley, *et al*.^35^. About 0.5 g of the dried sediment were treated with 10% HCl (T = 21 °C, 12 h) to remove carbonates and subsequently sieved using 25 µm nylon meshes. Using sodium polytungstate (SPT) as heavy liquid, the >25 µm fraction was split into three density fractions (<2.3 g/cm³, >2.3– <2.5 g/cm³, and >2.5 g/cm³). The two fractions >2.3 g/cm³ were mounted on microscope slides and the respective glass shard contents were counted using a polarization microscope (ZEISS AxioLab A1). Glass shard concentrations were calculated by normalizing the counted glass shard values for 1 g of dried sediment and used to determine the depositional characteristics of the cryptotephra (glass shard distribution vs. depth, isochrone position). Once the isochrone position of a cryptotephra was determined, a new aliquot of this sample interval was treated with 10% HCl, sieved with 25 µm mesh and if glass shard concentration were low, density separated using SPT as described above.

Bulk samples and respective processed aliquots of all macroscopic layers and of cryptotephra isochrone horizons were embedded in epoxy pucks. These pucks were polished to remove potential surficial alteration and to avoid topographic effects causing compositional variations. The pucks were carbon-coated for subsequent electron microbeam analysis. Identified tephra layers were labelled with the site name (OH-DP for Ohrid-DEEP) and the correlated bottom depth of each layer in decimeter (e.g., “OH-DP-0169”).

#### Single grain geochemical analysis

The geochemical fingerprint of a tephra layer was characterized by analyzing the geochemical composition of individual single grain glass fragments (glass shards, micropumices). Geochemical analysis included the analysis of major and minor element compositions of tephra layers by energy dispersive spectroscopy (EDS) and wavelength dispersive spectroscopy (WDS). Further, glass fragments of selected tephra layers were also analyzed for their trace element concentrations by Laser Ablation Inductively Coupled Plasma Mass Spectrometry (LA-ICP MS).

##### Major and minor element analysis (EDS/WDS)

Bulk tephra samples were inspected using an electron microscope (Philips XL30) at the Department of Geosciences of the University of Pisa (Italy)^36^. Quantitative major and minor element analyses on glass fragments were performed using the attached EDAX DX4 EDS system with operating conditions adjusted at 20 kV accelerating voltage, 200-500 nm beam diameter, 100 pA beam current and 100 sec live time with 2100-2400 shots per second. Depending on the available glassy area, a beam raster size of 5*5 or 10*10 µm was chosen, which was scanned by the beam accordingly. The EDAX DX4i software requires the analysis normalization at a given value, which is chosen at 100% and further enabled matrix corrections using the ZAF (Z-atomic number, A-absorption, F-fluorescence) correction procedure.

Tephra samples (bulk, aliquots and cryptotephra) were also measured by electron microprobe analyzer wavelength dispersive spectroscopy (EMPA-WDS) to increase the accuracy of quantitative analysis and to enlarge the comparability with existing reference data sets, of which most were obtained by EMPA-WDS. Samples were measured at the Istituto di Geologia Ambientale e Geoingegneria of the Italian National Research Council (IGAG CNR, Rome, Italy)^37^ and the University of Cologne (UoC, Germany)^38^.

Analysis at IGAG-CNR were performed with a Cameca SX50 electron microprobe equipped with five-wavelength dispersive spectrometers and operated with an accelerating voltage of 15 kV; a beam current of 15 nA, a beam diameter of 10 μm and a counting time of 20 sec per element. For calibration of the respective elements the following internal standards (magnetite (OXMT), fluor-phlogopite (MFSP), jadeite (PXYJD) from custom standard block of USGS all other minerals from P&H REE glass standards block, 1992) were used: wollastonite (Si, Ca), corundum (Al), periclase (Mg), magnetite (Fe), rutile (Ti), orthoclase (K), jadeite (Na), phlogopite (F), sylvite (Cl), baryte (S), apatite (P) and Mn (Mn-metal). Analyzed elements were apportioned on TAP (Na, Mg, Si, Al and F), PET (K, Ti, S, Ca, P, Cl) and LIF (Mn, Fe) analyzing crystals. Titanium contents were corrected for the spectral overlap of the Ti-Kα1 and Ti-Kα2 xray lines.

A JEOL JXA-8900RL electron microprobe equipped with five-wavelength dispersive spectrometers was used for analysis of glass fragments at the University of Cologne. The operation conditions were set to set to 12 kV accelerating voltage, 6 nA beam current and 5-10 µm beam diameter. Calibration of the machine was performed using the internal reference materials (Lipari glass ID3506, scapolite from the Smithsonian National Museum of Natural History and all other minerals from the P&H Geostandard Block), the analytical crystals and counting times: Lipari glass ID3506 (Si, Al; TAP; 20 sec), almandine (Fe, LIF; 30 sec), rutile (Ti; PETH; 30 sec), rhodenite (Mn; LIF; 30 sec), clinopyroxene (Mg; TAP; 30 sec), wollastonite (Ca; PETJ; 20 sec), albite (Na; TAP; 10 sec), orthoclase (K; TAP; 10 sec), apatite (P; PETH; 40 sec), scapolite NMNH R6600 (Cl; PETH; 40 sec), fluorite (F; TAP; 40 sec) and baryte (S; PETJ; 40 sec).

EPMA-WDS geochemical analyses of glass fragments normalized to 100% on a loss on ignition (LOI) free basis, excluding volatiles (Cl, SO_3_, and F). The tephra layers were classified according to their geochemical glass composition using the total alkali vs. silica (TAS) classification system^39^.

##### Trace element analysis (LA-ICP-MS)

Trace element analyses of selected tephra layers by LA-ICP-MS were performed on the same sample pucks as EPMA-WDS analysis. Tephra layers from the upper 247 mcd were analyzed at the Irish Center for Research in Applied Geosciences (iCRAG) at the Trinity College in Dublin (Ireland) and samples from 247-447 mcd at the University of Bonn (Germany).

At the iCRAG trace element laboratory in Dublin, a Teledyne Eximer 193 nm laser ablation system with a HelEx II two-volume ablation cell coupled to a Thermo Scientific iCapQ ICP-MS was used. A spot size of 18 µm, a repletion rate of 12 Hz and a count time of 40 sec (480 pulses) on the sample and 40 sec on the gas blank (background) were chosen. The ablated material was transported by He-N_2_ mixed gas flow (He 0.45 l*min^−1^, N_2_ 9.5 ml *min^−1^) via an inhouse signal smoothing device to the ICP-MS. Data reduction was performed in Iolite 3.5 using NIST SRM 612^40^ for calibration and ^29^Si (Si concentrations obtained by EPMA) as the internal standard for calculation of the trace element concentrations (Rb, Sr, Y, Zr, Nb, Ba, La, Ce, Pr, Nd, Sm, Eu, Gd, Dy, Er, Yb, Hf, Ta, Th, U). A secondary correction using Ca was applied as recommended by Tomlinson, *et al*.^41^.

At the University of Bonn trace element analyses of glass fragments were performed using a Resonetics Resolution M50E 193 nm excimer laser ablation system coupled to a Thermo Scientific Element XR. A spot size of either 15 or 20 µm was adapted to the grain specific available glassy areas and analyses were performed at a repetition rate of 5 Hz and a count time of 35 sec on the sample after 30 sec on the gas blank (background). A He gas flow (0.75 l* min^−1^), mixed together with Ar sample gas (~1.1 l* min^−1^) transported ablated material via an in-house signal-smoothing device to the ICP-MS. Maximum intensity as well as stability of the signal were obtained by tuning but taking account of concurrently low oxide ratios (ThO/Th of ~0.0012) to minimize potentially interfering oxide species prior to analyses in low-resolution mode. Data reduction was performed using the software Iolite 4.3 via calibration against NIST SRM 612^40^ and ^29^Si as the internal standard, with Si concentrations obtained by EPMA-WDS for calculation of trace element concentrations (Ca, Rb, Sr, Y, Zr, Nb, Cs, Ba, La, Ce, Pr, Nd, Sm, Eu, Gd, Tb, Dy, Ho, Er, Tm, Yb, Lu, Hf, Ta, Pb, Th, U)

## Data Records

The here presented datasets are stored at EarthChem (https://www.earthchem.org/, 10.26022/IEDA/112007)^42^ and individual files are described below. The structure of the datasheets is related to templates developed by the “tephra best practice team” and their recommendations in Abbott, *et al*.^43^. The dataset here presented includes information about position (core, core section depth, depth in composite succession), appearance (color, thickness and form of tephra layer, glass morphology), age (based on the age-depth model^2^) and glass geochemical composition (major, minor and trace elements) of 57 tephra layers identified in the DEEP site sediment succession from Lake Ohrid. In addition, raw-data of LA-ICP-MS measurements are given for each laboratory as.zip files. For each analytical method and each lab involved, files containing the specific methodological details are included. The entire dataset consists of previously published data, extensional data of these tephra layers and data of new tephra layers. Table 1 provides an overview of the data and its origin

### Tephra Overview Ohrid file

The overview files lists tephra layers and respective metadata presented within the dataset. Each row represents a tephra layer analyzed.

Label (column A): Label of tephra layer, consisting of the site name (OH-DP for Ohrid-DEEP) and the correlated bottom depth of each layer in decimeter (e.g., “OH-DP-0169”).

IGSN (column B): International Geo Sample Number, unique identification code of a sample provided by https://www.geosamples.org/.

Core (column C): Core from which the tephra was sampled. The core label includes the following information e.g., 1A-2H-1 = 1 A (drill site 1 and borehole A-E)-2H (number of drilling run and drilling tool used H = Hydraulic Piston Corer, E = Extended Nose Corer, A = Alien),−1 (number of section the recovered sediments was cut, usually a 3 m long drill run was split in three 1 m long sections).

Section Depth TOP (column D): The core section top depth in cm the tephra was sampled.

Section Depth BOTTOM (column E): The core section bottom depth in cm the tephra was sampled.

Composite Depth TOP (column F): The top depth of the tephra in the composite profile given in meters composite depth (mcd).

Composite Depth BOTTOM (column G): The bottom depth of the tephra in the composite profile given in meters composite depth (mcd).

Position of isochrone (column H): The isochrone depth in meters composite depth (mcd). The isochrone depth is the depth in which the tephra was originally deposited before it was e.g. affected by post sedimentary processes. The isochrone depth for macroscopic tephra layers is usually the bottom depth of this layer, which is determined on a visual basis. The isochrone of cryptotephra horizons is determined by glass shard concentration profiles, or is inferred from peaks in XRF downcore data.

Type of tephra (column I): The type of tephra is separated into macroscopic tephra (visible by naked eye) or cryptotephra (not visible by naked eye).

Form of tephra (column J): Describes the physical form of a tephra layer. Tephra described as “layer” are discrete sediment units spanning the entire core diameter. Tephra described as “lenses or patches” does not form a discrete layer and are only preserved as single patches or lenses. Tephra described as “invisible” are have no visible appearance and are cryptotephra.

Thickness (column K): Thickness of tephra layer in cm.

Color (column L): Macroscopic color of tephra, determined during visual core description.

Glass morphology (column L): Morphology of glass fragments determined during microscopic inspection and SEM analysis, following the classification of Katoh, *et al*.^44^.

SEM-EDS UoP (column N): “Yes” or “No”, if sample was analyzed by SEM-EDS at the University of Pisa.

EPMA-WDS IGAG CNR (column O): “Yes” or “No”, if sample was analyzed by EPMA-WDS at the Istituto di Geologia Ambientale e Geoingegneria of the Italian National Research Council.

EPMA-WDS UoC (column P): “Yes” or “No”, if sample was analyzed by EPMA-WDS at the University of Cologne.

LA-ICP-MS iCRAG TCD (column Q): “Yes” or “No”, if sample was analyzed by LA-ICP-MS at the iCRAG laboratory of the Trinity College Dublin.

LA-ICP-MS UoB (column R): “Yes” or “No”, if sample was analyzed by LA-ICP-MS at University of Bonn.

References datasets published (DOI) (column S-T): If data related to the sample was published elsewhere, the receptive DOI is given, otherwise samples described for the first time are marked as “this study”.

Extension of published datasets by the given type of data (column U): If the dataset of a sample was extended, the respective type of new data is given.

TAS-classification (column V): Classification of tephra layer according the Total Alkali vs. Silica diagram by Le Bas, *et al*.^39^.

Age (ka) (column W): Age of tephra layer according to age-depth model published in Wagner, *et al*.^2^.

### EPMA-SEM-EDS METHOD files

The analytical details such as the instruments and settings applied to analyze major and minor element concentrations by SEM-EDS and EPMA-WDS are given for each method and laboratory involved as a separate file^36–38^. The files are based on the EarthChem template and each file contains two parts, which is given in individual tabs. Note that only the columns of the template that are used are given below.

### EPMA-SEM Part 1 (tab 1)

Method DOI (column B): DOI to identify this method.

METHOD NAME (column C): Name of analytical protocol applied.

Method start date (column D): Date the protocol was used for the first time.

TECHNIQUE (column E): Analytical technique applied, either EPMA or SEM-EDS.

INSTRUMENT (column F): Instrument manufacturer and model.

LABORATORY (column G): Name of the laboratory and institution where the analyses were performed.

Laboratory ID (column H): ROR of the laboratory, if available (https://ror.org/)

Additional method references (column I): Other references in which this analytical protocol is described.

Funding (column J): Grant or other funding which supported the instrument used.

SOFTWARE (column L): Analytical and automation software used.

BEAM VOLTAGE (column M): Voltage of electron beam in kV.

BEAM CURRENT (column N): Current of electron beam in nA.

BEAM DIAMETER (column O): Beam diameter used with this method in µm.

BEAM RASTER (column P): If raster beam function was used (yes, none).

SECONDARY REFERENCE MATERIALS (column Q): List of quality control secondary reference materials used.

WDS utilization (column R): If wavelength dispersive spectrometry (WDS) was used. (yes or no).

WDS configuration (column S): Number and types of spectrometers used.

EDS utilization (column T): If energy dispersive spectrometry (EDS) was used. (yes or no).

EDS configuration (column U): Manufacturer and model of spectrometer used.

Beam damage minimization (column V): Approach to minimize for beam damage induced alkali migration.

X-ray matrix corrections (column X): X-ray matrix correction options selected in primary analytical software.

Additional notes (column Y): Any additional method details.

### EPMA-SEM Part 2 (tab 2)

Each row gives the settings for another parameter (column B) analyzed.

PARAMETER (column B): Measured element or oxide.

BEAM CURRENT (column D): Applied beam current in nA.

Spectrometer (column E): Type and number of spectrometer used

Sequence (column F): Order of analysis on spectrometer.

WDS Diffracting Crystal (column G): Type of WDS diffracting crystal used.

Detector Type (column H): Type of X-ray detector used.

X-ray Line (column I): X-ray line analyzed.

Peak Acquisition Time (seconds) (column J): Time in seconds used to analyze peak position.

Background Acquisition Method (column L): Method used for background acquisition.

WDS Background Acquisition Time (column M): Time in seconds used to analyze background position.

WDS PHA Setting (column N): Type of PHA settings chosen.

Primary Standard Name (column P): Reference material used for element calibration.

Primary Standard ID (column Q): Unique identification number of standard.

Detection Limit (column AA): Detection limit of element at 99% confidence.

Unit (column AB): Unit of detection limit.

Detection Limit Method (column AC): Method used to compute the method detection limits.

### LA-ICP-MS-METHOD files

The analytical details such as the instruments and settings applied to analyze trace element concentrations by LA-ICP-MS are given for each laboratory involved as a separate file^45,46^. The files are based on an EarthChem template and only the columns that are used are reported below.

Method DOI (column B): DOI to identify this method.

METHOD NAME (column C): Name of analytical protocol applied.

Method start date (column D): Date the protocol was used for the first time.

TECHNIQUE (column E): Analytical technique applied, either EPMA or SEM-EDS.

LABORATORY (column F): Name of the laboratory and institution the analyses were performed.

Laboratory ID (column G): ROR of the lab, if available (https://ror.org/)

Additional method references (column H): Other references in which this analytical protocol is described.

Funding (column I): Grant or other funding which supported the instrument used.

Laser system (column K): Laser system manufacturer and model.

Wavelength (column L): Wavelength of laser in nm

Repetition rate (column M): Repetition rate of laser in nm.

Fluence (column N): Energy density on target in J*cm^−^².

Pulse duration (column O): Duration of laser pulse in ns.

Laser spot size (column P): Spot size of laser in µm.

Automation Software (column Q): Software used for laser control and of spot selection.

Ablation Cell (column R): Type of ablation cell.

Cell gas flow (column S): Type and flux rate (ml *min^−1^) of gas used in ablation cell.

Trace gas flow (column T): Type and flux rate (ml *min^−1^) Used as trace gas.

Signal smoothing device (column U): Type of smoothing device.

Instrument (column V): Mass spectrometer manufacturer and model.

Type (column W): Type of mass spectrometer.

RF power (column W): Power of radio frequency signal in W.

Plasma gas flow (column W): Type of gas and flux rate (ml *min^−1^) of plasma gas flow.

Sample carrier gas flow (column W): Type of gas and flux rate (ml *min^−1^) of sample carrier gas flow.

Count time on sample (column W): Time in seconds counted on sample.

Count time on gas blank (column W): Time in seconds counted on gas blank before each sample.

Dwell time (column W): Dwell time in milliseconds.

Isotopes analyzed (column W): List of isotopes analyzed.

Calibration standards (column W): Name of standard used for calibration.

Sources for reference material concentration data (column W): Reference for reference values.

Internal standards (column W): Element chosen as internal standard.

Secondary standard (column W): List of secondary standards used for quality assessment.

Software (column W): Software used for data reduction.

Spline (column W): Type of spline used within data reduction.

DRS (column W): Name of data reduction scheme used.

Additional comments (column W): Additional comments

### EPMA-WDS_SEM-EDS Data Ohrid

The results of all geochemical analyses by SEM-EDS and EPMA-WDS of tephra layers and secondary reference standards are presented within this file. Each row labelled as “single” within column Z (data line type) represents the analysis of an individual glass fragment of the respective tephra layer or a single analysis of a secondary standard. Statistical values such as mean, standard deviation, respective relative bias to the preferred concentration in percent, and the relative standard deviation of the measurements are labelled in this column accordingly. Preferred values of secondary standards and the respective references are given along with the summarizing statistical values of the measurements. The secondary standard results associated to each analytical session can be linked based on the date and session ID given for the individual analyses. The file is based on the template developed by the recommendations of the tephra best practice team^43^ for the data repository EarthChem and provides general sample information in columns A-Z. The analyzed parameters normalized to 100% excluding volatiles (Cl, SO_3_, F) are given in columns AB:AO, whereas raw un-normalized values can be found in columns AS:BG. Please note that only the columns relevant for our study are used and explained below.

#### Sample ID (column B)

Label of tephra layer, consisting of the site name (OH-DP for Ohrid-DEEP) and the correlated bottom depth of each layer in decimeter (e.g., “OH-DP-0169”) or the respective name of the secondary standard.

#### IGSN (column C)

International Geo Sample Number, unique identification code of a sample provided by https://www.geosamples.org/.

Analyzed material (column D): Type of material analyzed: glass or mineral.

Analyzed as reference material? (Y/N) (column E): “Y”, if material was analyzed as reference material, or “N”, if material was analyzed as an unknown.

Sample Description (column F): Pretreatment of the sample analyzed (bulk = untreated sample material; wet sieved; respective density fraction).

Sample Mount Name (column G): Label used in laboratory for sample mount.

Data Point Notes (column H): Text to note quality of analysis and to mark e.g. outliers, possible mixed analyses (microcrysts), low analytical totals, etc.

Grain Number (column J): Number of each individual grain analyzed.

Laboratory (column R): Laboratory in which the analysis was performed (UoP = University of Pisa; IGAG CNR, Rome = Istituto di Geologia Ambientale e Geoingegneria of the Italian National Research Council Rome; UoC = University of Cologne).

Technique (column S): Type of instrument used for analyses (SEM-EDS; EPMA-WDS)

Method Protocol (column T): DOI number of the respective method file describing the analytical settings.

Analyst (column U): Analyst who has performed the analysis (GZ = Giovanni Zanchetta, RS = Roberto Sulpizio, BG = Biagio Giaccio, NL = Niklas Leicher) or the reference from which the preferred value was chosen.

Analysis Date and Time (column V): Date when the analysis was performed.

Analytical Session ID (column W): Session ID to identify each analytical session.

Beam Diameter (column X): Beam diameter of the instrument in µm used for spot analysis.

Number of Analyses (column Y): If data given as mean or standard deviation, the number of analyses is given.

Data Type Line (Z): Describes the type of data: single (single analysis), mean (mean of multiple analyses), standard deviation, relative standard deviation (in percent), bias% mean to reference value, preferred value (preferred value for secondary standard according to the reference given in column U.

Column AB-AN contain recalculated values in weight percent, normalized on a loss on ignition (LOI)-free basis (excluding Cl, SO_3_, F) to a total content of 100 wt.%, for *SiO*_*2*_ *(column AB), TiO*_*2*_ *(column AC), Al*_*2*_*O*_3_ *(column AD), FeO*_*t*_ *(column AE):* FeO_total_ (FeO + Fe_2_O_3_), *MnO (column AF), MgO (column AG), CaO (column AH), Na*_2_*O (column AI), K*_2_*O (column AJ), Cl (column AK), P*_2_*O*_5_ *(column AL), SO*_3_ *(column AM), F (column AN)* and the original analytical total (AO).

Analysis Line Number reported by the instrument software (column AQ): Label of analysed data point within the instrument software.

Columns AS-BG contain uncorrected analytical raw values of S*iO*_2_ *(column AS), TiO*_2_ *(column AT), Al*_2_*O*_3_ *(column AU), FeO*_*t*_ *(column AV):* FeO_total_ (FeO + Fe_2_O_3_), *MnO (column AW), MgO (column AX) CaO (column AY), Na*_2_*O (column AZ), K*_2_*O (column BA), Cl (column BB), P*_2_*O*_5_ *(column BC), SO*_3_ *(column BD), F (column BE), Analytical total (BF), Total volatile free (excl. Cl, SO3, F) (column BG)*.

### LA-ICP-MS Data Ohrid file

The results of all geochemical analyses by LA-ICP-MS of tephra layers and secondary reference standards are presented within this file. Each row labelled as “single” within column Z (data line type) represents the analysis of an individual glass fragment of the respective tephra layer or a single analysis of a secondary standard. Statistical values such as mean, standard deviation, respective relative bias to the preferred concentration in percent, and the relative standard deviation of the measurements are labelled in column Z accordingly. Preferred values and associated uncertainties of secondary standards and the respective references are given accordingly. The secondary standard results associated to each analytical session can be linked based on the time stamp given for the individual analyses. The file is based on the template developed by the recommendations of the tephra best practice team^43^ for the data repository EarthChem and provides general sample information in columns A-Z. The analyzed parameters are given in columns AB:BA, whereas respective element specific propagated 2 standard errors (2SE int) are given in BG:CF. The 2SE int was calculated within the Iolite software (version 4.3, Paton, *et al*.^47^). Please note that only the columns relevant for our study are used and explained below.

*Sample ID (column B):* Label of tephra layer, consisting of the site name (OH-DP for Ohrid-DEEP) and the correlated bottom depth of each layer in decimeter (e.g., “OH-DP-0169”) or the respective name of the secondary standard.

*IGSN (column C):* International Geo Sample Number, unique identification code of a sample provided by https://www.geosamples.org/.

Analyzed material (column D): Type of material analyzed: glass or mineral.

Analyzed as reference material? (Y/N) (column E): “Y”, if material was analyzed as reference material, or “N”, if material was analyzed as an unknown.

Sample Description (column F): Pretreatment of the sample analyzed (bulk = untreated sample material; wet sieved; respective density fraction).

Sample Mount Name (column G): Label used in laboratory for sample mount.

Data Point Notes (column H): Text to note quality of analysis and to mark e.g. outliers, possible mixed analyses (microcrysts), low analytical totals, etc.

Grain Number (column J): Number of each individual grain analyzed.

*Laboratory (column R):* Laboratory in which the analysis was performed (iCRAG Trinity College Dublin or University of Bonn)

Technique (column S): Type of instrument used for analyses (LA-ICP-MS)

Method Protocol (column T): DOI number of the respective method file describing the analytical settings.

Analyst (column U): Analyst who has performed the analysis (ET = Emma Tomlinson, PA = Paul Albert, ML = Markus Lagos, NL = Niklas Leicher) or the reference from which the preferred value was chosen.

Analysis Date and Time (column V): Date when the analysis was performed.

Analytical Session ID (column W): Session ID to identify each analytical session.

Beam Diameter (column X): Beam diameter of the instrument in µm used for spot analysis.

Number of Analyses (column Y): If data given as mean or standard deviation, the number of analyses is given.

Data Type Line (Z): Describes the type of data: single (single analysis), mean (mean of multiple analyses), standard deviation, relative standard deviation (in percent), bias% mean to reference value, preferred value (preferred value for secondary standard according to the reference given in column U.

The trace element concentrations of analyzed glass fragments are given in columns AB:BA. Ca (µg/g) *(column AB)*, Rb (µg/g) *(column AC)*, Sr (µg/g) *(column AD)*, Y (µg/g) *(column AE)*, Zr (µg/g) *(column AF)*, Nb (µg/g) *(column AG)*, Ba (µg/g) *(column AH)*, La (µg/g) *(column AI)*, Ce (µg/g) *(column AJ)*, Pr (µg/g) *(column AK)*, Nd (µg/g) *(column AL)*, Sm (µg/g) *(column AM)*, Eu (µg/g) *(column AN)* Gd (µg/g) *(column AO)*, Tb (µg/g) *(column AP)*, Dy (µg/g) *(column AQ)*, Ho (µg/g) *(column AR)*, Er (µg/g) *(column AS)*, Tm (µg/g) *(column AT)*, Yb (µg/g) *(column AU)*, Lu (µg/g) *(column AV)*, Hf (µg/g) *(column AW)*, Ta (µg/g) *(column AX)*, Pb (µg/g) *(column AY)*, Th (µg/g) *(column AZ)*, U (µg/g) *(column BA)*. The element specific 2 standard error (2SE int) calculated in Iolite based on Paton, *et al*.^47^ is given in columns BG:CF in the same order the elements are given above.

## Technical Validation

### Chronology of the DEEP site record

Ages of tephra layers were inferred from the age-depth model of the DEEP site sediment record^2^ based on the combination of tie points from tephrochronology and orbital tuning. Tephra ages from literature were only used if geochemical correlations were unambiguous and respective chronological uncertainties of ages were precise ( < 6 ka) as discussed in detail in Wagner, *et al*.^2^^,^ and Leicher, *et al*.^23^. ^40^Ar/^39^Ar ages of sanidines or leucites of equivalent tephra layers were recalculated using the same decay constant^48^ and the same ages for the mineral standards Alder Creek sanidine-2 (ACs-2) of 1.1891 Ma^49^ and for the Fish Canyon sanidine of 28.294 Ma^48^. Chronological tie points obtained by orbital tuning were treated with an error of ± 2 ka, accounting for inaccuracies in data processing and tuning^2^.

The resulting chronology was evaluated by a comparison with the U/Th dated Soreq Cave (0-160 ka) and the Pequiin Cave (180-250 ka) speleothem records and the orbitally tuned Tenaghi Philippon pollen record (1.36 Ma)^2^. The results were also validated by a comparison with paleomagnetic age constraints of the succession (base of Jaramillo subchron and Matuyama Brunhes reversal, see also Just, *et al*.^50^), suggesting an agreement with all chronologies within errors^2^.

The intrinsic age uncertainty of analyzed tephra layers is inferred from the Bayesian age-depth model (Bacon2.2^34^) on a 95% confidence interval, assuming a normal/ Gaussian probability distribution for tephra and orbital tie points (normal = TRUE).

### Geochemical analysis

Besides the analysis of secondary reference materials to ensure quality of measurements, further steps were considered to assess data quality. EPMA-WDS geochemical analyses of glass fragments were classified based on their total analytical sum and only analysis with totals exceeding 65% were reported. Results with totals between 65 and 90% were classified as “low total results”, whereas only analysis with totals > 90% were considered as reliable results. Based on the broad range of data given, individual cutoff values can be applied when reusing the data and adjusted e.g. to different degrees of glass hydration. The number of EPMA-WDS analyses were chosen to be statistically relevant with generally n ≥ 20, if not limited by the number of glass shards present. This allowed detection and depiction of the natural variability of the glass composition (e.g. magma heterogeneity and differentiation, post sedimentary mixture/reworking), but also helped to detect analytical outliers or misperformance of the instruments. The results were also screened for the influence of phenocrysts during measurements and analyses were excluded, if respective influence was detected. Some samples were measured in all laboratories involved using both SEM-EDS and EPMA-WDS techniques, which enables an additional assessment of data quality and to characterize offsets between the different laboratories and techniques involved. However, this approach has to be seen as a first-order approximation as the samples do have a natural variability in composition. Among the samples analyzed with all techniques applied, three of them show a relatively narrow homogeneous composition, promoting a comparison of the mean of the individual measurements. The data confirm the general performance of individual methods and settings applied as no significant offsets can be noticed for concentrations above 0.1 wt.% (Online-only Table 1). Individual differences between the laboratories are reported along with the method and laboratory specific data assessment below.

#### SEM-EDS analysis

For SEM-EDS analysis of the University of Pisa, secondary standard data is not available on a session related basis to evaluate individual analytical performance of the machine. However, the general long term performance of the machine was documented by analyzing different reference standards (albite and olivine^51^ and the glasses CFA47^52^ and KE12^53^) during multiple sessions. These data suggest a mean analytical precision (RSD%) and accuracy (bias of mean to preferred reference value in %) of 0.3 and 0.5% respectively for element abundances > 15 wt.%, 1.4 and 0.9% for 15-5 wt.%, 2.9 and 4.5% for 5-1 wt.% and 29.3 and 20.1% for < 0.6 wt.%. These results are in line with earlier tests performed by Marianelli and Sbrana^51^ reporting a precision of 0.5% for abundances higher than 15 wt.%, 1% for abundances around 5 wt.%, 5% for abundances of 1 wt.%, and less than 20% for abundances close to the detection limit around 0.5 wt.%. Based on the inter-laboratory comparison of three tephra layers analyzed in all labs, SEM-EDS results differ from EPMA-WDS results as summarized in Online-only Table 1. SEM-EDS results of SiO_2_, Al_2_O_3_, MgO are higher and TiO_2_, FeO, MnO, Na_2_O, Cl are lower compared to those obtained by EPMA-WDS analysis at the UoC. CaO and K_2_O concentrations do not differ systematically as values are higher and lower. Comparing SEM-EDS with EPMA-WDS results obtained at the IGAG-CNR Al_2_O_3_, MgO and K_2_O are higher, whereas SiO_2_, TiO_2_, FeO, MnO and Cl are lower. Results for CaO and Na_2_O do not differ systematically and can be both higher and lower.

#### EPMA-WDS analysis

All analyses are marked with a respective analytical session ID, which allows linking the results of session specific standard analyses with the associated sessions of unknown samples (e.g. tephra samples). The EPMA laboratory at IGAG used two international secondary standards (Kakanui augite USNM 122142^54^ and rhyolite RLS132 glass^55^ from the United States Geological Survey) prior to sample measurements to evaluate data quality. The mean analytical precision (RSD%) and accuracy (bias of mean to preferred reference value in %) are respectively 0.3-0.9% and 0.3-0.4% for element abundances > 50 wt.%, 0.8-1.6% and 0.5-3.5% for element abundances between 20-10 wt.%, 0.1-2.5% and 0.4-3.8% for element abundances between 8-4 wt.%, up to 1.7-4.7% and 5.9-10.6% for element abundance 2-1 wt.%; up to 6.3-30% and 0.9-13.8% for element abundance 1.0-0.1 wt.% and up to 31% (both) for element abundance < 0.1wt.%.

During microprobe analysis at the UoC, MPI-DING glasses (ATHO-G; StHs6/80-G; GOR132-G; GOR128-G)^56^ were used as secondary standards to evaluate the accuracy and precision of measurement sessions. Individual results of MPI-DING glasses are provided in the dataset for each measurement session along with mean values for precision (RSD%) and accuracy (bias%). General mean values for precision and accuracy of analyses are respectively both 1.0% for elemental concentrations > 40 wt.%, 3.3% and 2.4% for 25-5 wt.%, 8.9% and 2.4% for 5-1 wt.%, 27.6% and 12.7% for 1-0.2 wt.% and 74% and 10% for 0.2- 0.1 wt.%.

For the evaluation of potential alkali loss (e.g. Na_2_O loss), the given preferred values of reference standards have to be carefully assessed. The preferred value for Na_2_O in ATHO-g given by Jochum, *et al*.^56^ is about 3.75 wt.%, but appears to be too low as methods such as LA-ICP-MS and XRF, which are not affected by electron beam induced alkali loss, indicate higher concentrations of about 4.26-4.61 wt.%^56,57^. Data obtained within the EPMA-WDS lab of the UoC supports this observation with 312 out of 441 analyses above 3.75 wt.%, having a mean of 4.2 wt.% for Na_2_O. Also the analysis of other, less beam sensitive secondary standard glasses (e.g. StHs6/80-G, GOR-128) suggest that Na_2_O loss does not have excessively occurred, and thus secure the general performance of the applied settings. However, as the other 129 analyses of ATHO-g indicate lower values, Na_2_O-loss cannot fully be excluded for all analytical sessions and thus should be considered than reusing the data.

The inter-laboratory comparison (Online-only Table 1) suggests that the EPMA-WDS concentrations obtained at IGAG-CNR are higher for SiO_2_ and MgO and lower for FeO, Mn, Na_2_O relative to the ones obtained at the UoC. Concentrations of TiO_2_, Al_2_O_3_, CaO and Cl do not show a systematic deviation, as values are observed to be higher and lower.

#### LA-ICP-MS analyses

All analyses are marked with a respective session ID, which allows linking the results of session specific standard analysis with the associated sessions of unknown samples (e.g. tephra samples). Secondary standard analyses of MPI-DING glass (ATHO-G, StHs6/80-G, GOR132-G)^56^ during measurement sessions at the iCRAG lab of the Trinity College in Dublin revealed mean accuracies of 4-8% for Rb, Sr, Zr, Ba, Nd, Sm, Eu and Th, of 8-10% for La, Ce, Pr, Gd, Dy and Yb and of 10-15% for Y, Nb, Er, Hf, Ta and U. The mean precision of the MPI-DING glass analyses were typically ≤ 5% relative standard deviation (RSD) for Rb, Sr, Y, Zr, Ba, Ce, Nd, Sm, Eu, Gd, Dy, Er and Th and 5-8% for Nb, La, Pr, Yb, Hf, Ta and U.

At the University of Bonn (UoB), secondary standard analyses of MPI-DING (ATHO-G, StHs6/80-G, GOR132-G, KL2-G, ML3B-G, T1-G^56^), USGS (BHVO-2G^58^) and SRM NIST SRM 610^40^ glasses revealed mean accuracies of 4-8% for Ca, Rb, Sr, Ba, La, Ce, Pr, Sm, Tb, Ho, and Tm, 8-10% for Y, Nd, Eu, Dy, Er, Yb, Lu, Hf, Th, and U and 10-12% for Zr, Nb, Gd, Ta and Pb. The mean precision of the secondary standard analyses were typically < 5% relative standard deviation (RSD) for Ca, Rb, Sr, Y, Zr, Nb, Ba, La, Ce and Pr, and of < 10% for Nd, Sm, Eu, Gd, Dy, Ho, Pb, and Th and 10-18% for Tb, Er, Tm, Yb, Lu, Hf, Ta, and U. The influence of the two applied spot sizes on the performance of the analyses was evaluated considering the same analyzed standards (NIST610, ATHO-G) and is summarized in Online-only Table 2. The mean values of NIST610 obtained using a 20 µm spot size are between 0.3% and 5.6% higher compared to the mean values of 15 µm spot size analysis for all elements except for Hf, which is about 2.6% lower. The relative biases of the 15 µm spot size analysis to the preferred concentrations of NIST 610^40^ are between 0.4% and 5.4% lower (Online-only Table 2) than those of the 20 µm spot size analyses, with the same exception for Hf. Considering the means of ATHO-G analyses, the differences between the spot sizes are not systematically higher towards the 20 µm spot size analysis as observed for NIST610. However, the general magnitude of variations is in a similar range than those of NIST610 analysis and 20 µm spot size analyses vary relative to 15 µm analysis between 0.1 and 6.3% for all elements except for Eu, which is 9.8% higher. With regard to the bias relative to the preferred concentrations of ATHO-G^56^, 20 µm spot size trend to be more accurate with some exceptions, which do not follow a systematic trend such as e.g. mass fractionating (cf. Online-only Table 2).

A comparison of the analytical performance of the iCRAG TCD with the UoB laboratory is based on the standards ATHO-G and StHs6/80-G^56^ analyzed in both laboratories and is given in Online-only Table 2. Mean values of both standards suggest that for most elements the obtained concentrations are between 0.03% and 17.3% lower in the UoB laboratory compared to the iCRAG results, with some irregular exceptions showing higher values (StHs6/80-G: Gd, Dy, Er; ATHO-G: Gd; cf. Online-only Table 2). Comparing the laboratory specific bias relative to the preferred concentrations of the standards reveals that the iCRAG TCD results are more accurate relative to StHS6/80-G for all elements except for Dy, and for most elements relative to ATHO-G, except for Ba and Gd (Online-only Table 2).

## Online-only Tables

**Online-only Table 1:** Comparison of SEM-EDS and EPMA-WDS results.

Sample ID	Laboratory	Technique		SiO_2_ (wt.%)	TiO_2_ (wt.%)	Al_2_O_3_ (wt.%)	FeO_t_ (wt.%)	MnO (wt.%)	MgO (wt.%)	CaO (wt.%)	Na_2_O (wt.%)	K_2_O (wt.%)	Cl (wt.%)	P_2_O_5_ (wt.%)	SO_3_ (wt.%)	F (wt.%)	Original Total (wt.%)
OH-DP-1700.6	UoP	SEM-EDS	mean (n = 7)	73.62	0.05	14.51	0.95	0.01	0.33	1.30	2.98	6.24	0.18				100.00
OH-DP-1700.6	UoP	SEM-EDS	standard deviation	0.22	0.06	0.15	0.06	0.04	0.08	0.10	0.21	0.20	0.03				0.01
OH-DP-1700.6	UoP	SEM-EDS	relative standard deviation	0.3%	125.1%	1.0%	6.1%	244.9%	23.0%	7.9%	7.1%	3.1%	17.8%				0.0%
OH-DP-1700.6	IGAG CNR	EPMA-WDS	mean (n = 11)	73.82	0.13	14.51	1.10	0.06	0.08	1.16	3.14	5.99	0.22	0.01	0.01	0.42	95.81
OH-DP-1700.6	IGAG CNR	EPMA-WDS	standard deviation	0.17	0.03	0.11	0.05	0.02	0.02	0.05	0.07	0.08	0.02	0.01	0.01	0.17	1.33
OH-DP-1700.6	IGAG CNR	EPMA-WDS	relative standard deviation	0.2%	20.2%	0.8%	4.3%	25.8%	19.5%	4.1%	2.4%	1.3%	7.7%	130.3%	105.7%	42.0%	1.4%
OH-DP-1700.6	UoC	EPMA-WDS	mean (n = 17)	73.59	0.13	14.24	1.10	0.12	0.08	1.02	3.17	6.52	0.23	0.03	0.02	0.43	95.13
OH-DP-1700.6	UoC	EPMA-WDS	standard deviation	0.82	0.03	0.32	0.14	0.06	0.04	0.10	0.70	0.40	0.01	0.02	0.02	0.23	0.99
OH-DP-1700.6	UoC	EPMA-WDS	relative standard deviation	1.1%	21.2%	2.2%	13.1%	51.8%	53.3%	9.7%	22.1%	6.1%	5.8%	83.3%	127.7%	52.1%	1.0%
			*BIAS% UoP to UoC*	*0.03%*	*−60.18%*	*1.90%*	*−13.68%*	*−88.09%*	*334.37%*	*27.01%*	*−5.96%*	*−4.25%*	*−21.27%*				
			*BIAS% UoP to IGAG CNR*	*−0.28%*	*−61.41%*	*0.02%*	*−13.36%*	*−75.74%*	*289.80%*	*12.32%*	*−5.06%*	*4.21%*	*−19.21%*				
			*BIAS% IGAG CNR to UoC*	*0.31%*	*3.20%*	*1.88%*	*−0.37%*	*−50.90%*	*11.43%*	*13.07%*	*−0.94%*	*−8.12%*	*−2.55%*	*−60.50%*	*−25.17%*	*−4.16%*	
OH-DP-2603	UoP	SEM-EDS	mean (n = 20)	63.15	0.41	18.77	2.63	0.19	0.56	1.06	6.64	6.61	0.41				100.00
OH-DP-2603	UoP	SEM-EDS	standard deviation	0.28	0.09	0.35	0.09	0.09	0.09	0.08	0.46	0.23	0.06				0.01
OH-DP-2603	UoP	SEM-EDS	relative standard deviation	0.4%	22.7%	1.9%	3.2%	49.6%	16.0%	7.6%	7.0%	3.5%	14.2%				0.0%
OH-DP-2603	IGAG CNR	EPMA-WDS	mean (n = 19)	64.48	0.47	18.46	2.63	0.21	0.40	1.11	5.83	6.37	0.47	0.06	0.10	0.29	95.91
OH-DP-2603	IGAG CNR	EPMA-WDS	standard deviation	0.46	0.03	0.15	0.09	0.04	0.03	0.05	0.36	0.12	0.04	0.02	0.02	0.08	0.93
OH-DP-2603	IGAG CNR	EPMA-WDS	relative standard deviation	0.7%	5.6%	0.8%	3.6%	17.2%	6.7%	4.1%	6.3%	1.9%	9.2%	33.1%	22.5%	25.9%	1.0%
OH-DP-2603	UoC	EPMA-WDS	mean (n = 47)	61.95	0.48	18.61	2.69	0.25	0.39	1.14	7.79	6.63	0.42	0.07	0.10	0.28	94.64
OH-DP-2603	UoC	EPMA-WDS	standard deviation	0.41	0.03	0.24	0.18	0.08	0.03	0.07	0.29	0.19	0.03	0.03	0.05	0.21	0.78
OH-DP-2603	UoC	EPMA-WDS	relative standard deviation	0.7%	7.3%	1.3%	6.7%	33.5%	8.5%	6.0%	3.7%	2.9%	6.6%	52.1%	48.0%	75.8%	0.8%
			*BIAS% UoP to UoC*	*1.93%*	*−15.15%*	*0.85%*	*−1.91%*	*−25.56%*	*43.48%*	*−7.53%*	*−14.81%*	*−0.43%*	*−4.40%*				
			*BIAS% UoP to IGAG CNR*	*−2.06%*	*−12.28%*	*1.69%*	*0.00%*	*−12.19%*	*40.18%*	*−4.64%*	*13.91%*	*3.76%*	*−13.34%*				
			*BIAS% IGAG CNR to UoC*	*4.07%*	*−3.27%*	*−0.82%*	*−1.91%*	*−15.22%*	*2.36%*	*−3.03%*	*−25.21%*	*−4.04%*	*10.32%*	*−12.61%*	*−1.82%*	*5.91%*	
OH-DP-2669	UoP	SEM-EDS	mean (n = 16)	63.39	0.38	18.96	2.36	0.08	0.72	1.22	5.92	6.97	0.31				100.00
OH-DP-2669	UoP	SEM-EDS	standard deviation	1.13	0.13	0.46	0.37	0.11	0.19	0.22	0.58	0.95	0.13				0.01
OH-DP-2669	UoP	SEM-EDS	relative standard deviation	1.8%	34.9%	2.4%	15.7%	140.5%	26.3%	18.3%	9.8%	13.7%	43.6%				0.0%
OH-DP-2669	IGAG CNR	EPMA-WDS	mean (n = 20)	64.10	0.41	18.47	2.55	0.19	0.41	1.30	5.73	6.76	0.40	0.07	0.15	0.21	95.99
OH-DP-2669	IGAG CNR	EPMA-WDS	standard deviation	0.72	0.05	0.28	0.14	0.04	0.10	0.23	0.30	0.32	0.11	0.03	0.04	0.09	0.53
OH-DP-2669	IGAG CNR	EPMA-WDS	relative standard deviation	1.1%	13.0%	1.5%	5.5%	21.0%	25.1%	17.8%	5.2%	4.8%	27.1%	52.1%	26.1%	41.5%	0.5%
OH-DP-2669	UoC	EPMA-WDS	mean (n = 69)	62.95	0.41	18.92	2.62	0.22	0.40	1.07	6.55	6.80	0.39	0.06	0.13	0.28	97.14
OH-DP-2669	UoC	EPMA-WDS	standard deviation	0.51	0.05	0.37	0.21	0.09	0.10	0.20	0.56	0.53	0.11	0.04	0.05	0.21	1.50
OH-DP-2669	UoC	EPMA-WDS	relative standard deviation	0.8%	12.0%	1.9%	8.0%	41.6%	24.6%	19.2%	8.5%	7.9%	27.6%	66.6%	40.9%	73.7%	1.5%
			*BIAS% UoP to UoC*	*0.70%*	*−8.75%*	*0.18%*	*−9.84%*	*−65.28%*	*80.97%*	*14.29%*	*−9.63%*	*2.57%*	*−19.79%*				
			*BIAS% UoP to IGAG CNR*	*−1.11%*	*−8.93%*	*2.62%*	*−7.20%*	*−60.99%*	*73.72%*	*−6.52%*	*3.35%*	*3.17%*	*−22.06%*				
			*BIAS% IGAG CNR to UoC*	*1.83%*	*0.20%*	*−2.38%*	*−2.84%*	*−10.99%*	*4.17%*	*22.26%*	*−12.56%*	*−0.58%*	*2.91%*	*3.71%*	*14.54%*	*−24.14%*	

Based on three samples, which have been each analyzed by SEM-EDS at the University of Pisa (UoP), EPMA-WDS at the IGAG CNR in Rome and EPMA-WDS at the University of Cologne (UoC), the analytical performance of the different techniques and laboratories is assessed. For each laboratory and sample the mean, the standard deviation and relative standard deviation of the laboratory individual analyses is given. The percentagewise difference (bias%) of the means relative to the individual laboratories allows assessment of differences in the analytical performance.

**Online-only Table 2:** Comparison of analytical performances of LA-ICP-MS systems.

Standard	Laboratory	Beam Diameter (µm)		Ca (µg/g)	Rb (µg/g)	Sr (µg/g)	Y (µg/g)	Zr (µg/g)	Nb (µg/g)	Ba (µg/g)	La (µg/g)	Ce (µg/g)	Pr (µg/g)	Nd (µg/g)	Sm (µg/g)	Eu (µg/g)	Gd (µg/g)	Tb (µg/g)	Dy (µg/g)	Ho (µg/g)	Er (µg/g)	Tm (µg/g)	Yb (µg/g)	Lu (µg/g)	Hf (µg/g)	Ta (µg/g)	Pb (µg/g)	Th (µg/g)	U (µg/g)
**Comparison 15 µm and 20 µm spotsizes UoB**																													
*ATHO-G*	*Jochum et al., 2006*		*preferred value*	*12150*	*65.3*	*94.1*	*94.5*	*512*	*62.4*	*547*	*55.6*	*121*	*14.6*	*60.9*	*14.2*	*2.76*	*15.3*	*2.51*	*16.2*	*3.43*	*10.3*	*1.52*	*10.5*	*1.54*	*13.7*	*3.9*	*5.67*	*7.4*	*2.37*
*ATHO-G*	*Jochum et al., 2006*		*preferred value uncertainty*	*214*	*3*	*2.7*	*3.5*	*20*	*2.6*	*16*	*1.5*	*4*	*0.4*	*2*	*0.4*	*0.1*	*0.7*	*0.08*	*0.7*	*0.11*	*0.5*	*0.07*	*0.4*	*0.05*	*0.5*	*0.2*	*0.62*	*0.27*	*0.12*
ATHO-G	UoB	20	mean (n = 13)	12118	62.8	93.2	87.9	477	54.8	532	53.3	117	14.4	57.4	14.1	2.8	14.7	2.4	15.5	3.4	9.9	1.5	10.0	1.5	13.3	3.4	5.2	7.2	2.2
ATHO-G	UoB	20	standard deviation	363	0.8	1.6	1.6	21	2.7	8	1.0	3	0.2	2.6	0.9	0.3	0.7	0.1	0.5	0.2	0.7	0.1	0.5	0.1	0.5	0.1	0.3	0.3	0.1
ATHO-G	UoB	20	bias mean to preferred value%	-0.3%	-3.8%	-1.0%	-7.0%	-6.9%	-12.1%	-3%	-4.1%	-3%	-1.7%	-5.7%	-0.7%	1.0%	-4.2%	-4.1%	-4.1%	-2.0%	-4.4%	-2.6%	-5.0%	-4.3%	-3.0%	-11.8%	-8.3%	-2.8%	-8.3%
ATHO-G	UoB	20	relative std. deviation	3.0%	1.2%	1.8%	1.8%	4.3%	4.9%	1%	1.8%	2%	1.6%	4.6%	6.2%	12.1%	5.0%	5.3%	3.2%	5.6%	7.5%	7.1%	5.0%	6.5%	3.7%	3.9%	4.9%	4.2%	4.9%
ATHO-G	UoB	15	mean (n = 24)	12578	63.3	90.8	85.7	463	55.4	522	52.0	116	13.8	57.5	13.3	2.5	14.8	2.3	15.2	3.3	9.7	1.5	10.1	1.5	12.5	3.3	5.1	6.9	2.2
ATHO-G	UoB	15	standard deviation	2047	3.4	2.8	3.6	23	1.9	22	1.9	3	0.6	3.9	1.5	0.3	2.7	0.3	1.9	0.4	1.8	0.2	1.2	0.2	2.2	0.3	0.6	0.4	0.2
ATHO-G	UoB	15	bias mean to preferred value%	3.5%	-3.1%	-3.5%	-9.3%	-10%	-11.1%	-5%	-6.4%	-4%	-5.7%	-5.6%	-6.1%	-8.1%	-3.1%	-6.9%	-6.0%	-5.1%	-6.1%	-1.8%	-4.1%	-5.1%	-8.7%	-14.7%	-10.0%	-6.5%	-6.2%
ATHO-G	UoB	15	relative std. deviation	16.3%	5.4%	3.1%	4.1%	5%	3.5%	4%	3.6%	3%	4.7%	6.7%	11.4%	11.4%	18.4%	11.5%	12.4%	10.8%	18.1%	15.1%	12.3%	13.4%	17.4%	8.9%	12.0%	5.3%	10.3%
ATHO-G			bias% mean 20 to 15 µm	-3.7%	-0.8%	2.6%	2.6%	2.9%	-1.1%	2.0%	2.5%	1.2%	4.3%	-0.1%	5.9%	9.8%	-1.2%	3.0%	2.1%	3.3%	1.9%	-0.7%	-1.0%	0.9%	6.3%	3.3%	1.9%	4.0%	-2.2%
*NIST610*	*Jochum et al., 2011*		*preferred value*	*81475*	*425.7*	*515.5*	*462*	*448*	*465*	*452*	*440*	*453*	*448*	*430*	*453*	*447*	*449*	*437*	*437*	*449*	*455*	*435*	*450*	*439*	*435*	*446*	*426*	*457.2*	*461.5*
*NIST610*	*Jochum et al., 2011*		*preferred value uncertainty*	*1429*	*1*	*1*	*11*	*9*	*34*	*9*	*10*	*8*	*7*	*8*	*11*	*12*	*12*	*9*	*11*	*12*	*14*	*10*	*9*	*8*	*12*	*33*	*1*	*1*	*1*
NIST610	UoB	20	mean (n = 38)	83769	436.7	524.3	472	457	480	463	455	468	467	443	470	461	458	458	448	464	465	444	471	458	445	465	435	474.0	476.7
NIST610	UoB	20	standard deviation	3685	25.9	36.4	33	36	33	32	36	34	39	42	37	33	39	34	46	39	40	32	35	39	45	40	41	41.8	37.2
NIST610	UoB	20	bias mean to preferred value%	2.8%	2.6%	1.7%	2.2%	2.1%	3.3%	2.5%	3.5%	3.3%	4.3%	3.0%	3.7%	3.2%	2.0%	4.8%	2.5%	3.3%	2.3%	2.2%	4.7%	4.3%	2.4%	4.3%	2.1%	3.7%	3.3%
NIST610	UoB	20	relative std. deviation	4.4%	5.9%	6.9%	7.0%	7.8%	6.9%	6.8%	8.0%	7.3%	8.4%	9.4%	7.9%	7.2%	8.5%	7.5%	10.3%	8.3%	8.6%	7.1%	7.5%	8.6%	10.2%	8.7%	9.3%	8.8%	7.8%
NIST610	UoB	15	mean (n = 31)	81850	433.0	514.7	460	444	478	455	441	460	450	434	447	453	440	442	436	449	441	434	463	435	457	452	422	459.6	463.7
NIST610	UoB	15	standard deviation	3348	22.5	13.7	12	23	14	20	9	14	15	16	17	22	19	14	15	14	31	12	27	14	88	13	15	12.9	14.2
NIST610	UoB	15	bias mean to preferred value%	0.5%	1.7%	-0.1%	-0.4%	-1%	3%	1%	0.1%	2%	0.4%	1%	-1%	1%	-2%	1%	-0.1%	0.1%	-3%	-0.2%	3%	-1%	5%	1%	-1%	0.5%	0.5%
NIST610	UoB	15	relative std. deviation	4.1%	5.2%	2.7%	2.6%	5.1%	3.0%	4.3%	2.1%	3.0%	3.2%	3.7%	3.8%	4.8%	4.3%	3.2%	3.5%	3.1%	7.1%	2.9%	5.7%	3.3%	19.3%	2.9%	3.6%	2.8%	3.1%
NIST610	UoB		bias% mean 20 to 15 µm	2.3%	0.9%	1.8%	2.7%	3.0%	0.3%	1.7%	3.3%	1.6%	3.9%	2.0%	5.0%	1.8%	4.0%	3.6%	2.6%	3.3%	5.6%	2.4%	1.7%	5.3%	-2.6%	2.8%	3.1%	3.1%	2.8%
**Comparison University of Bonn // iCRAG Trinity College Dublin**																													
*StHs6_80-G*	*Jochum et al., 2006*		*preferred value*	*37736*	*30.7*	*482*	*11.4*	*118*	*6.94*	*298*	*12.0*	*26.1*	*3.20*	*13.0*	*2.78*	*0.953*		*0.371*	*2.22*	*0.420*	*1.18*	*0.172*	*1.13*	*0.168*	*3.07*	*0.420*	*10.3*	*2.28*	*1.01*
*StHs6_80-G*	*Jochum et al., 2006*		*preferred value uncertainty*	*643*	*1.7*	*8*	*0.4*	*3*	*0.25*	*9*	*0.3*	*0.7*	*0.06*	*0.3*	*0.05*	*0.022*	*0.09*	*0.011*	*0.06*	*0.011*	*0.04*	*0.007*	*0.03*	*0.006*	*0.09*	*0.015*	*0.9*	*0.07*	*0.04*
StHs6_80-G	UoB	20 + 15	mean (n = 28)	34529	29.1	443	10.2	105	5.86	274	10.8	23.3	2.88	11.6	2.59	0.770	3.00	0.384	2.12	0.398	1.29	0.164	1.01	0.177	2.61	0.374	8.2	2.05	0.86
StHs6_80-G	UoB	20 + 15	standard deviation	3149	1.8	13.25	0.83	8.00	0.59	13.18	0.74	1.00	0.27	1.82	0.64	0.25	1.38	0.19	0.53	0.12	0.54	0.20	0.59	0.10	0.84	0.13	0.89	0.23	0.15
StHs6_80-G	UoB	20 + 15	bias mean to preferred value%	-8.5%	-5.3%	-8.1%	-10.1%	-11.4%	-15.6%	-8.2%	-10.3%	-10.9%	-10.0%	-10.4%	-6.7%	-19.2%	15.7%	3.5%	-4.3%	-5.2%	9.7%	-4.6%	-10.8%	5.1%	-14.9%	-10.9%	-20.2%	-10.2%	-14.5%
StHs6_80-G	UoB	20 + 15	RSD%	9.1%	6.3%	3.0%	8.1%	7.7%	10.1%	4.8%	6.9%	4.3%	9.5%	15.6%	24.5%	33.0%	46.1%	49.3%	25.1%	30.6%	42.1%	124.8%	58.8%	55.7%	32.2%	34.6%	10.8%	11.4%	17.6%
StHs6_80-G	iCRAG TCD	18	mean (n = 19)		31.0	490	10.5	112	6.84	313	11.9	26.2	3.04	12.9	2.81	0.931	2.41		2.05		1.12		1.08		2.73	0.403		2.20	1.01
StHs6_80-G	iCRAG TCD	18	standard deviation		1.58	18.32	0.73	5.39	0.35	14.35	0.74	1.74	0.14	0.43	0.20	0.05	0.15		0.19		0.11		0.12		0.21	0.04		0.15	0.09
StHs6_80-G	iCRAG TCD	18	bias mean to preferred value%		1.0%	2%	-7.7%	-5%	-1.4%	5%	-0.6%	0.5%	-5.0%	-0.8%	1.3%	-2.3%	-6.9%		-7.6%		-5.5%		-4.3%		-11.0%	-3.9%		-3.5%	-0.5%
StHs6_80-G	iCRAG TCD	18	relative std. deviation		5.1%	4%	6.9%	5%	5.0%	5%	6.2%	6.6%	4.5%	3.3%	7.3%	5.7%	6.2%		9.1%		9.5%		10.7%		7.8%	9.5%		6.9%	8.6%
			bias% UoB to iCRAG TCD		-6.3%	-10%	-2.6%	-7%	-14.3%	-13%	-9.7%	-11.4%	-5.3%	-9.7%	-7.9%	-17.3%	24.2%		3.6%		16.0%		-6.8%		-4.4%	-7.2%		-6.9%	-14.1%
*ATHO-G*	*Jochum et al., 2006*		*preferred value*	*12150*	*65.3*	*94.1*	*94.5*	*512*	*62.4*	*547*	*55.6*	*121*	*14.6*	*60.9*	*14.2*	*2.76*	*15.3*	*2.51*	*16.2*	*3.43*		*1.52*	*10.5*	*1.54*	*13.7*	*3.9*	*5.67*	*7.4*	*2.37*
*ATHO-G*	*Jochum et al., 2006*		*preferred value uncertainty*	*214*	*3.0*	*2.7*	*3.5*	*20*	*2.6*	*16*	*1.5*	*4*	*0.4*	*2.0*	*0.4*	*0.10*	*0.7*	*0.08*	*0.7*	*0.11*	*0.5*	*0.07*	*0.4*	*0.05*	*0.5*	*0.2*	*0.62*	*0.3*	*0.12*
ATHO-G	UoB	20 + 15	mean (n = 37)	12445	63.2	91.6	86.5	468	55.4	525	52.4	116	13.9	57.4	13.6	2.64	14.8	2.36	15.3	3.29	9.7	1.49	10.1	1.47	12.8	3.4	5.1	7.0	2.20
ATHO-G	UoB	20 + 15	std. deviation	1677	2.8	2.7	3.2	23	2.3	19	1.7	3	0.6	3.5	1.4	0.33	2.2	0.23	1.6	0.31	1.5	0.19	1.0	0.17	1.8	0.3	0.5	0.4	0.20
ATHO-G	UoB	20 + 15	bias mean to preferred value%	2.4%	-3.2%	-2.6%	-8.5%	-8.7%	-11.2%	-4.0%	-5.7%	-3.9%	-4.5%	-5.7%	-4.5%	-4.5%	-3.1%	-5.8%	-5.3%	-4.0%	-5.5%	-1.8%	-4.2%	-4.7%	-6.9%	-13.8%	-9.2%	-5.7%	-7.1%
ATHO-G	UoB	20 + 15	relative std. deviation	13%	4%	3%	4%	5%	4%	4%	3%	3%	4%	6%	10%	12%	15%	10%	10%	9%	15%	13%	10%	11%	14%	8%	10%	5%	9%
ATHO-G	iCRAG TCD	18	mean (n = 20)		66.0	97.0	88.3	495	61.0	570	56.2	125.0	14.9	62.3	14.7	2.70	14.2		16.2		9.7		10.1		12.8	3.9		7.38	2.36
ATHO-G	iCRAG TCD	18	std. deviation		3.2	6.2	7.1	31	2.2	35	3.9	7.9	0.9	3.6	1.2	0.18	1.1		1.2		0.9		0.8		1.2	0.3		0.52	0.11
ATHO-G	iCRAG TCD	18	bias mean to preferred value%		1.0%	3.1%	-6.6%	-3%	-2.3%	4%	1.1%	3%	2.1%	2.4%	3.8%	-2.1%	-7.2%		-0.1%		-5.4%		-3.8%		-6.8%	-0.8%		-0.27%	-0.50%
ATHO-G	iCRAG TCD	18	relative std. deviation		4.8%	6.4%	8.0%	6%	3.6%	6%	6.9%	6%	6.1%	5.7%	8.1%	6.8%	7.5%		7.5%		9.6%		8.3%		9.4%	6.9%		7%	5%
			bias% UoB to iCRAG TCD		-3.4%	-4.9%	0.2%	-4.6%	-9.5%	-8.0%	-6.1%	-6.9%	-6.2%	-7.4%	-7.1%	-1.8%	5.4%		-3.1%		2.4%		1.7%		2.4%	-13.8%		-3.9%	-6.2%

The upper part of the table shows a comparison of secondary standard values measured using different spot sizes during analytical sessions at the University of Bonn (UoB). The mean values for measurements of ATHO-G^57^ and NIST610^40^ are given for analyses performed using 20 and 15 µm laser spot sizes and are compared with the recommended standard values. For each standard, the percentwise difference (bias%) between the 20 µm spot size relative to the 15 µm spot size results is given. The second part of the table shows a comparison of the general analytical performance of the LA-ICP-MS systems of the UoB and the iCRAG TCD laboratories based on results obtained for secondary standards ATHO-G and StHs6_80-G57. Individual laboratory results are compared with the respective recommended values and a percentwise difference (bias%) between the UoB results relative to the iCRAG TCD results is given.
